# Remote control catheter navigation: options for guidance under MRI

**DOI:** 10.1186/1532-429X-14-33

**Published:** 2012-06-01

**Authors:** Leah Muller, Maythem Saeed, Mark W Wilson, Steven W Hetts

**Affiliations:** 1 Department of Radiology and Biomedical Imaging, University of California San Francisco, 505 Parnassus Avenue, L-352, San Francisco, CA, 94143-0628, USA

**Keywords:** Interventional MRI, Remote control catheter guidance

## Abstract

**Background:**

Image-guided endovascular interventions have gained increasing popularity in clinical practice, and magnetic resonance imaging (MRI) is emerging as an attractive alternative to X-ray fluoroscopy for guiding such interventions. Steering catheters by remote control under MRI guidance offers unique challenges and opportunities.

**Methods:**

In this review, the benefits and limitations of MRI-guided remote control intervention are addressed, and the tools for guiding such interventions in the magnetic environment are summarized. Designs for remote control catheter guidance include a catheter tip electromagnetic microcoil design, a ferromagnetic sphere-tipped catheter design, smart material-actuated catheters, and hydraulically actuated catheters. Remote control catheter guidance systems were compared and contrasted with respect to visualization, safety, and performance. Performance is characterized by bending angles achievable by the catheter, time to achieve bending, degree of rotation achievable, and miniaturization capacity of the design. Necessary improvements for furthering catheter design, especially for use in the MRI environment, are addressed, as are hurdles that must be overcome in order to make MRI guided endovascular procedures more accessible for regular use in clinical practice.

**Conclusions:**

MR-guided endovascular interventions under remote control steering are in their infancy due to issues regarding safety and reliability. Additional experimental studies are needed prior to their use in humans.

## Background

Image-guided endovascular interventions have gained increasing popularity in clinical practice as studies have shown consistently that these minimally invasive interventions are of equivalent or greater efficacy and confer lower morbidity when compared to traditional open surgical techniques [1-8]. A majority of interventions involve the use of a flexible catheter that is guided by the interventionalist into the appropriate vessels [9] under real-time X-ray fluoroscopy imaging. However, while X-ray fluoroscopy provides clear navigation in patent vessels and allows high spatial and temporal resolution, there are drawbacks to its use (such as lack of soft tissue visualization), making magnetic resonance imaging (MRI) an attractive alternative for remotely steering catheters during endovascular interventions. Although prior limitations to MRI guided interventions have been overcome in the last decade, including the development of near real-time dynamic MR “fluoroscopic” imaging sequences, steering catheters within blood vessels remains challenging as compared to steering of catheters under X-ray guidance. The use of MRI for endovascular catheter navigation is a growing field of study with significant clinical promise, and many innovative techniques for guiding a catheter in the magnetic field of the MRI scanner are being proposed and tested [10,11]. The development of these augmented catheter guidance techniques stands to break down one of the most significant barriers to adoption of MRI as a real-time interventional guidance modality.

### Potential advantages of MRI for guiding interventions

A clear advantage of using real-time MRI for intervention lies in the wealth of physiologic and structural information provided by the MR image itself. By visualizing the soft tissue surrounding a blood vessel as opposed to only the vessel lumen, the interventionalist may assess the function of an organ as a procedure is performed [11]. For example, in the case of treating an acute ischemic stroke caused by thromboembolic occlusion of a cerebral artery, it is possible to visualize the ischemic penumbra (via MR perfusion imaging) surrounding the core infarct (via MR diffusion weighted imaging) such that clinical determination of whether to reopen an occluded artery can occur while the intervention is being executed [12]. This real-time evaluation of tissue damage could prevent hemorrhage upon reopening an artery into brain tissue that is already infarcted [12]. Additionally, MR perfusion and thermometry allow for monitoring the effects of procedures such as thermal and cryo-ablations [13]. Real-time tissue visualization is useful in guiding a variety of interventions, including, for example: tumor embolization, aneurysm occlusion, angioplasty, and myocardial stem cell delivery [9].

MRI has at least two potential advantages over X-ray fluoroscopy that may increase safety for the patient and the physician. First, MRI does not necessarily involve the injection of iodinated contrast, which has been associated with complications, including nephrotoxicity and anaphylaxis [14]. Although contrast-enhanced MRA involves the use of gadolinium or blood pool agents, noncontrast MRA techniques allow vascular visualization without the attendant risks for contrast administration. Second, whereas X-ray fluoroscopy uses significant doses of potentially damaging ionizing radiation, MRI uses only lower energy, non-ionizing radiation that has no known long term deleterious health consequences [13].

### Challenges for MRI guidance of interventions

MRI has its own challenges that must be overcome before it is used as a first line real-time intervention guidance system. Traditional devices and robotic systems designed for use in operating rooms and X-ray fluoroscopy suites that use electromagnetic components, such as actuators and sensors, are rendered useless or dangerous in the clinical high magnetic field scanners. A device incompatible with the magnetic environment produces susceptibility artifact that can negatively affect image quality [15]. Additionally, the long, narrow bore of many MR scanners dictates that any tool used in combination with MRI must be compact or attached to electronic controllers outside the 5 Gauss line of the MR scanner’s fringe field [15].

### Challenges for endovascular catheter navigation

Maneuverability of a catheter for intravascular navigation in any imaging environment is key to reaching the target area; ability to steer a catheter thus affects to a great extent the length and success of the procedure. Difficulty in steering and control of the catheter increase the risk for complications, including vascular dissection, perforation, and thrombosis [16]. Some of these risks can be offset by systemic heparinization, which is in routine use in clinical endovascular procedures performed today, though may itself increase procedural hemorrhage risk. For many procedures the catheter is guided from a safe entrance vessel (e.g., common femoral artery) to a target that is relatively far away in the body via branching or tortuous vessels. With a traditional catheter, the catheter tip is directed by manually rotating the catheter about its axis and pushing it forward into the desired vessel, often over a variably stiff coaxially placed guidewire. After the catheter has been navigated through several vascular turns, the torque at the proximal end is hindered [16] and control of the guidewire tip is limited [9]. Additionally, it is difficult to manipulate a catheter tip through sharp turns, for instance entering a recurrent branch vessel whose origin is directed at a greater than 90 degree orientation to the parent vessel [9].

## Catheter navigation techniques and approaches

### Guidewires and pullwire catheters

With these evident challenges, various guidance mechanisms for catheter navigation have been designed and are in clinical use. Manual and pullwire guidance were the first to be used. Guidewires for manual direction are flexible, small diameter wires placed through the patent lumens of catheters and are manually navigated into branch vessels before the functional catheter tip to serve as a stable track for the catheter to follow [17]. Manually controlled variable stiffness shapeable metallic guidewires placed through variably stiff plastic catheters have been the mainstay of endovascular catheter guidance under X-ray fluoroscopic imaging for decades, and the number of guidewires and catheters available for specific applications throughout the body is huge. MR compatible coaxial guidewires and catheters are few and far between, however, given the ferrous metals often used in the wire cores or braided in the walls of the catheters.

The pullwire system was first introduced as an encasement at the tip of the catheter consisting of two wires on opposite sides of a helical spring with a leaf spring on one side to provide rigidity. When one of the wires is pulled, the catheter tip deflects in that direction [16]. Improvements on this design include the use of two sets of orthogonal pull wires for movement in two planes and two sets of parallel pull wires for bending the catheter tip in an S-curve shape [16]. A further improvement on this design is an adjusting sleeve that may slide up the length of the deflecting portion of the catheter tip for lengthening or shortening the part of the catheter that bends in response to pull wire stimulation. The pullwire catheters still have limited range and flexibility, and size is a limiting factor such that navigation into small vessels is limited [16]. Similar to guidewires for use in X-ray angiographic environment, springs in pullwire systems are often made of materials not compatible with or safe for the MR environment.

A current example of an electromechanical robotic system for catheter direction and specifically for intracardiac ablation is the Sensei (Hansen Medical, CA, USA). This device makes use of a master/slave system using a coaxial catheter with two guiding sheaths of 14.0 F and 11.5 F, which are used cooperatively in the device as an inner and outer steering sheath. Smaller catheters less than 8.5 F diameter are inserted through the guide catheter to reach the target tissue, as the guiding sheaths can shape into various configurations with the help of pull wires in the inner sheath [18]. This system has the disadvantage of its diameter and size being limiting factors on how far it can reach into small vessels, as well as the curve radius of the sheaths being a limitation to the turning radius of the catheter [18]. This method has been used with X-ray fluoroscopy, and is not compatible with MR imaging due to the ferromagnetic metallic materials used.

### Magnetic navigation in the X-ray fluoroscopy environment

Magnetic navigation presently can be performed in the operating room or X-ray angiography suite using the Niobe system (Stereotaxis, St. Louis, MO), which consists of two large permanent magnets positioned around the operating table that create a magnetic field of 0.08 T to guide a magnetically tipped microguidewire for a catheter through vessels under real-time X-ray fluoroscopy. The external magnets are rotated and angled to change the orientation of the field and therefore of the position of the catheter tip [19]. Software for this system includes imaging and point-and-click navigation tools, and most systems are integrated with a C-arm X-ray fluoroscopic imaging system [20]. A technical challenge for the system is aligning the virtual image obtained from a preoperative planning MRI with the real-time image obtained from X-ray fluoroscopy during the procedure, as almost inevitably there are tissue shifts due to patient repositioning between the time of MRI and the time of the procedure [20]. Time can also be an issue, as each magnetic field manipulation requires 5 seconds to activate [19]. Because the Niobe system uses large external permanent magnets, it is inherently incompatible with real-time use of MRI in procedure guidance.

## Magnetic navigation in the MRI scanner

### Catheter tip microcoil approach

**Figure 1:**
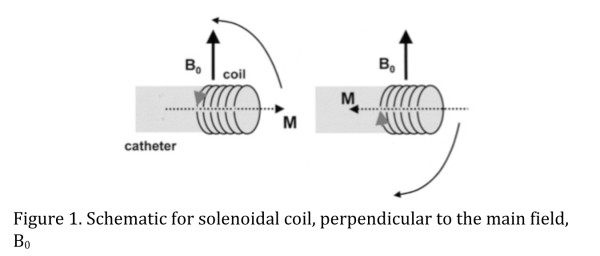
**Schematic for solenoidal coil, perpendicular to the main field,B**_**0**_**.**

While the magnetic Niobe system is inherently incompatible with MRI scanners, the use of the MR environment itself presents a unique opportunity for catheter guidance. Roberts et al. prototyped a magnetically-assisted remote control catheter design that uses coils of copper to create a magnetic moment when energized [10]. The concept of this design is that a solenoidal coil generates a magnetic moment when electric current is passed through it. In the presence of a strong magnetic field, like the 1.5 T field generated by standard MRI, the coil and catheter tip experience a turning force, or torque, to align the small magnetic moment of the coil with the external field of the MR scanner [10]. By changing the polarity of the current, the catheter tip can either align or anti-align with the external field [10] (Figure 1).

**Figure 2:**
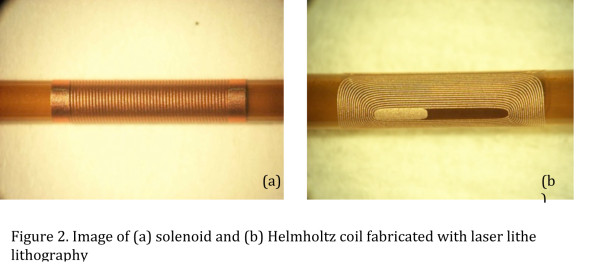
Image of (a) solenoid and (b) Helmholtz coil fabricated with laser lithe lithography.

The original design consisted of a solenoid coil of copper wound around a standard catheter tip. This has been improved to allow more planes of movement by adding two additional coils to the solenoid, creating a design that layers a solenoid coil underneath two orthogonal modified Helmholtz coils, or paired “racetrack” windings [10] (Figure 2). With three independent coils capable of deflecting in three orthogonal fields, the tip of the catheter may be oriented by remote control in 3D space within the body, simply by selectively energizing one or more coils [10]. The coils at the tip are created using laser lathe lithography, a technique that allows nonplanar surfaces such as cylinders to be patterned with feature sizes as small as 5 μm. Coil designs are patterned onto standard size catheter tips using a photoresist and electroplated copper for the coil material. Since the relative force or torque is determined by the magnetic field created, the number of turns of the solenoid or racetrack coil can be manipulated to produce a magnetic field that is more or less powerful depending on the force needed to turn the catheter tip [21]. The catheter coil need only be energized for a brief time period, on the order of seconds, to achieve deflection for navigation into the desired vessel [21]. Because the coils create a miniature magnetic field, there is an observable artifact at the catheter tip that may be used to actively visualize the catheter tip when it is energized [10]. When no current is run through the catheter tip coils minimal or no image artifact is present, allowing high-resolution local tissue imaging.

### Catheter tip ferromagnetic sphere approach

**Figure 3:**
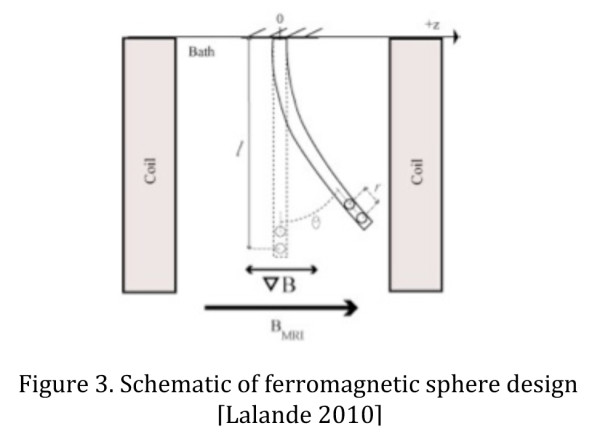
Schematic of ferromagnetic sphere design [Lalande 2010].

Another technique using magnetic forces to guide a catheter inside the MR field is using a catheter tip equipped with ferromagnetic spheres that can be controlled by varying magnetic field gradients. The spheres are made of chrome steel and are enclosed in a rigid casing to allow for free rotation [22]. One and two magnetic spheres were tested, and it was shown that a deflection of the catheter tip was produced in any desired direction with gradient changes produced by custom Maxwell coils inserted into the bore of the scanner [23]. (Figure 3) By changing the distance separating two spheres, larger tip deflections may be achieved with minimal dipole-dipole interactions. However, this length of separation needed between the spheres may also limit the turning radius of the catheter tip. The spheres produce a large artifact due to distortion of the local magnetic field inside the scanner, and thus are problematic for imaging tissue directly surrounding the catheter tip [22].

### Smart material approach

Smart material actuators provide an alternative to magnetic and metallic guidance, with the added benefit that there is no interference with the magnetic environment using this method of actuation. Shape memory polymers (SMPs) are made by a cycle of heating, shaping, cooling, and fixing such that the unit can be stored in a temporary shape and triggered to reshape into its former design [24]. Direct heat is most commonly used to trigger an SMP to resume its original shape, although ultraviolet and infrared radiation, electric current, pH changes, and magnetic field have also been used. In a study by Buckley, inductive heating was used to induce a change in SMP shape by loading ferromagnetic particles into the polymer and exposing it to an alternating magnetic field, as could be used in conjunction with MRI [25]. Shape memory alloys (SMAs) are better studied than SMPs and have a shorter response time, but are higher density, higher cost, and have lower attainable strains [26]. A shape memory alloy actuated catheter has been designed such that tubes of SMA are distributed about the central axis of the catheter and can be heated to achieve bending due to shrinking of the SMA material [16]. When heating stops, the material is naturally cooled and reforms its original shape [16]. Some major considerations with these methods are safe heating, efficient cooling, and the use of many lead wires if the catheter assumes a multi-link style [16]. At least some shape memory alloys are MRI compatible and thus may find useful application in MRI guided procedures [27].

### Hydraulic approach

Hydraulic catheters share the advantage of having no interference with the MR scanner, as they are driven by fluid pressure in tubes on the sides of the catheter. Increasing pressure bends the catheter away from the pressurized tube [16]. Miles patented a pneumatic or hydraulic catheter design in which tubing runs on either side of a catheter to an elastomeric cylindrical catheter tip in which one or more steering lumens are offset from the longitudinal axis of the catheter [28]. In a design such as this, catheter direction could be elicited by using a pneumatic or hydraulic pressure source or by heating a thermally expandable material filling the steering lumens [28]. The need for multiple lumens does increase catheter size, and may limit this design for microcatheters.

### Comparison and analysis

Each of the described methods of catheter navigation within the MR environment has benefits and drawbacks. Three main categories stand out in discussing these catheter designs in comparison to each other: visualization, safety, peripheral nerve stimulation, tissue damage, and steering performance.

### Visualization

**Figure 4:**
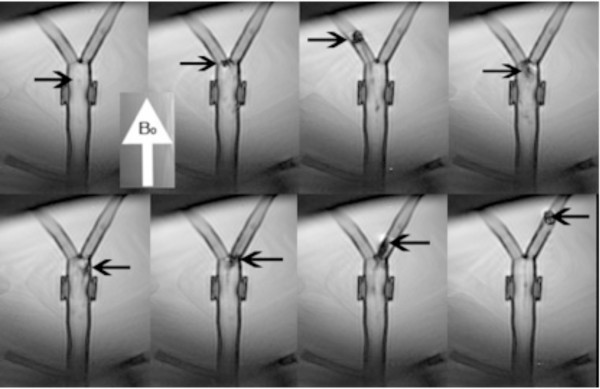
**Navigation of magnetic catheter under steady state free precession real-time imaging in an aortic bifurcation phantom with B**_**0**_**indicating the direction of the MR scanner bore.** The upper left panel demonstrates minimal image artifact from the copper-coil tipped catheter (arrow) when no electric current is applied. When a small positive electric current is applied to the catheter tip (second upper panel), the catheter deflects to the left and then can be pushed into the left branch vessel (third upper panel) and then withdrawn to the simulated aorta (fourth upper panel). When a small negative current is applied to the catheter tip (lower panels), the catheter is deflected to the right and selects the right branch vessel.

Visualizing the catheter tip while navigating vessels is important so that the catheter tip is not forced undesirably through a vessel or tissue, causing perforation, and so that it may be directed efficiently within vessels to reach a target (Figure 4). In terms of visualization, the two options that use magnetic fields, either created by electric current as in the coil design or by amplified fields and magnetic beads, will inevitably distort the image due to interference. However, it should be noted that this artifact produced could be useful in visualizing the catheter tip if it is not too overwhelming in the image. With the coil tipped catheter design, charging the coils with current produces a miniature magnetic field; thus a visual artifact is present only when the coils are charged for navigation [21]. A smaller ‘visualization’ current could be used to intentionally produce an artifact on a smaller scale for a longer time period while maneuvering the catheter through vessels [29]. Alternatively, catheter tip coils could receive MR RF signals for active determination of catheter tip position, similar to previously described active catheter tracking techniques [10]. In the ferromagnetic sphere design, there is an inherent artifact due to the ferromagnetic material comprising the spheres; however, this artifact is smaller when a single sphere is used rather than two spheres, and even with two spheres a single artifact is produced because the spacing is close between spheres [22]. For the hydraulic and smart material actuated catheters, the catheter itself may produce a visualization artifact if ferromagnetic materials are used, and for the hydraulic catheter, the hydraulic fluid could be doped with a contrast agent.

### Safety

For the coil tipped catheter design, both resistive heating and radiofrequency (RF) heating must be considered. Heat dissipation is a major concern for applications in living tissue; the standards by the US Food and Drug Administration for recommended temperature rise should not exceed 1°C on or in the head and 2°C in the torso and extremities [30]. Studies on the coil catheter design show that resistive heating was ameliorated to an acceptable temperature range for intervention by adopting an alumina catheter tip over the original polyimide material and by running room temperature saline through the lumen of the catheter, similar to current clinical practice in which saline drips are used to reduce the risk of catheter thrombosis [21]. RF heating concerns center around the ‘antenna effect’ in which RF magnetic fields used for MRI coupled with a long wire such as a guidewire, even if not ferromagnetic, causes local heating near the device [31]. Preliminary studies on RF heating of the coil device show that no clinically significant RF-induced heating occurs during MR imaging under the tested conditions [31]. Given that other metal-containing devices have undergone unexpected RF induced heating during MR guided interventions [32], further testing is warranted.

Heat used to activate SMAs or SMPs is intentionally applied, and should be maintained below the FDA standards while still eliciting an effectual change in the catheter shape. This means that the material used should have a large enough thermal expansion coefficient that a small deliberate change in temperature can elicit the desired change in shape; this required specificity can make designing such a device challenging. Very focal delivery of energy to joints or other points of actuation may mitigate this limitation.

### Peripheral nerve stimulation and tissue damage

Another consideration for safety regards the use of high amplitude magnetic gradients in the ferromagnetic sphere design. To avoid peripheral nerve stimulation caused by powerful gradients with fast switching rates, the rate of change of the magnetic gradient should be kept below 20 T/s [22]. This limit is easily maintained for regular catheter steering times in an interventional procedure [22]. Vessel or tissue perforation with force of bending of the catheter is always a concern; however, none of the aforementioned MRI guidance techniques report any higher propensity for perforation than the manually guided catheters in use today; moreover, the new designs may improve safety due to better and more predictable steering. Manual feedback to the hand of the interventionalist on the hub end of a catheter would also provide an additional level of safety, similar to that in current clinical practice.

### Steering performance

Performance is the major consideration in designing a remotely steerable catheter. Performance is characterized in this paper by bending angles achievable by the catheter, time to achieve bending, degree of rotation achievable, and miniaturization capacity of the design.

An equation for predicting deflection and experimental validation of this equation have been studied for the coil tipped catheter. The equation is based on the number of solenoid turns, applied current, catheter stiffness, and magnetic field strength [9]. The magnetic torque created by energizing the coils causes catheter deflection up to the point that deflection is balanced by the mechanical restoring torque of the catheter [9]. It was found that a linear relationship exists between the angular mechanical deflection and the number of solenoid turns, and also that a doubling in field strength (1.5 T to 3 T in MRI scanner) resulted in a doubling of angular mechanical deflection over a wide range of current values [9]. For clinical consideration, a standard 1.8 F catheter tip of 7 mm length, enhanced with a solenoid of 100 turns, in a 1.5 T environment and charged with 300 mA current could deflect up to 90° [9]. To minimize length, a lesser number of turns and a higher current could have the same effect. Of note, the catheter tip can be held by magnetic force on a specific target for over one minute, which may be useful for ablation applications [9]. The time to achieve bending is nearly instantaneous, so that the coil-tipped catheter could be charged for a few seconds to navigate into a vessel [9]. With three orthogonal coils [33], the coil-tipped catheter can navigate in almost any direction. This design is produced using laser lithography, so it has the capacity to be miniaturized onto catheter tips with dimensions as small as 5 μm. Limitations on miniaturization of diameter include sheathing one coil inside another, so possible alternative designs have been suggested for spreading coils along the length of the catheter tip to achieve very small diameters [33].

The ferromagnetic sphere design has the advantage of allowing movement in any direction independent of the main MRI magnetic field through the use of magnetic gradient forces [22]. As mentioned, the tip may be equipped with one or two ferromagnetic beads, and it has been shown that configurations with two beads attain deflections approximately twice as large as those with one bead. However, bending achieved with two spheres produces an S-shaped curve in the catheter tip that grows more pronounced with increased distance between the beads. The largest deflection achieved in one study was 20.3 mm lateral movement of the tip with two beads spaced 4.5 mm apart and with free tip length of 32.7 mm [22]. A maximum degree of deflection was shown in a figure to be approximately 50 degrees [23]. Deflection of this amplitude comes with the added bulkiness of a second bead and casing at the tip of the catheter. An additional concern is that with only one bead or with two beads spaced too closely, the tip of the catheter ‘jumps’ unpredictably in amplitude, precluding precise control of the catheter. The time to achieve a deflection is based on the time to ramp up the magnetic gradient, which is nearly instantaneous [22].

SMPs and SMAs include a variety of materials that could be used in augmenting catheter navigation, each having different properties of thermal conductivity and thermal expansion. In general, SMPs take tens of seconds to recover their original shape, while SMAs take tens of milliseconds [26]. Cooling to rebound to original shape is generally slow with SMAs, since forced cooling cannot occur in such a small structure [16]. Additionally, SMAs exhibit high hysteresis and non-linearity, making their precision questionable [16]. Miniaturization is easily conceivable with this device; however, this requires a relatively small amount of SMA or SMP material be responsive to actuation.

The hydraulic or pneumatic catheter proposed would have a rapid response time to changes in fluid or gas pressure [16]. Depending on the flexibility of the catheter material surrounding the pressurizing lumens, this design could achieve a comparable turning radius to a pullwire catheter. Precision and force control could be a problem in such a device, for pneumatic and hydraulic actuators alike express nonlinearity caused by slight differences in gas compressibility, flow characteristic at the entrance valve, and friction losses by the actuator [15]. Additionally, parameters of these systems are easily affected by temperature, shape, friction, and fatigue, and thus are changeable during a procedure [15]. The referenced design can be readily miniaturized while still preserving a lumen for functional catheter applications [28].

## Future directions

Because perforation and dissection when navigating vessels or treating tissue are safety concerns when performing an intervention, a pressure sensor at the tip of the catheter is desirable. This desire must be weighed against the additional bulk that such sensors would add to catheter tips, as well as the potential for RF heating of additional conducting wires. Thus, larger catheters for use in larger blood vessels are easier to conceptualize than microcatheters for use in small cerebral or coronary vessels. To be compatible with MRI, the options for such a pressure sensor are somewhat limited, but include aluminum force sensors [34] and fiber optics [15].

Temperature sensing is an essential component of many procedures, such as thermal and cryo-ablation. While MR thermometry is within the capability of the scanner and could be used to obtain a general idea of temperatures within tissues, it is limited by interference from blood flow or other movement and by local distortion of the magnetic field by ferromagnetic components of the catheter. A more specific temperature sensor at the catheter tip would enable safer use of the catheter for interventions in which the tip is heated or cooled, either to affect a target tissue as in ablation, or to induce a change in catheter orientation for navigation. This temperature sensor must be able to be miniaturized so not to add bulkiness to the catheter; thermocouples are an obvious first step to achieving temperature sensing on a small scale.

For performance purposes, steerable MR-compatible catheters that include a specialized catheter tip should be designed to achieve the shortest length tip possible so that the modification does not in itself limit too harshly the turning radius of the catheter tip. Limitation of turning radius occurs if the length of coil needed to generate sufficient magnetic moment for turning is too long to make the turn into a vessel. To fully utilize the capability of MRI for real-time navigation and soft tissue imaging, any device to navigate a catheter in an MR scanner should be operable without having to pause imaging for an extended period of time. For many designs, this combination of control and visualization may be achieved through a compromise in which signals to elicit a change in position of the catheter are interleaved with signals that give feedback and images describing the anatomy. Finally, an intuitive control system by which the interventionalist may operate the device and thereby efficiently guide the catheter to the target is crucial to the realization of faster and safer catheterization for intervention under MR guidance.

## Review and conclusions

Remote control catheter navigation for MRI guided interventional endovascular procedures may become an attractive alternative to X-ray fluoroscopic navigation due to MRI’s soft tissue and physiologic imaging capabilities, its relative safety for both interventionalist and patient, and its unique opportunity for magnetically-actuated catheter tip steering in the context of MR imaging. New devices for catheter navigation are being devised to address the demand for tools that are compatible with the magnetic environment. These devices are varied in the materials and technique used for navigation, and each has benefits and drawbacks (Table 1) that should be taken into account in future designs, particularly with regard to steering performance, safety, and visualization.

**Table 1:** Comparison of Remote Control Mechanisms for Catheter Tip Steering

**Remote Control Mechanism**	**Strengths**	**Weaknesses**
Catheter Tip Microcoils	· Minimal image distortion when current is off	· Current carrying wires in catheter may undergo RF heating
	· Microcoils can act as receiver coils for active imaging	· Microcoils may undergo resistive heating
		· Multilayer coils add bulk and stiffness to catheter tip
Catheter Tip Ferromagnetic Beads	· Low potential for catheter tip heating	· Image distortion by beads may be difficult to eliminate
	· Simple design improves manufacturability	· Separation between beads may make tip long and rigid
		· Possible peripheral nerve stimulation by gradients used for steering
		· Discontinuous jumps in tip deflection
Smart Material Actuators	· Easily miniaturizable	· May be difficult to deliver enough heat to change tip shape without heating adjacent tissue
		· Prolonged cooling times to return to original shape
		· High hysteresis and non-linearity limit deflection precision
Hydraulic Catheter	· Could add contrast agent to hydraulic fluid for better visualization	· Susceptible to temperature changes and fatigue during long procedures, limiting reliability
	· Easily miniaturizable	

## Competing interests

Drs. Hetts, Wilson, and Saeed have received grant funding from the National Institutes of Health (1R01EB012031-01A1 Hetts, PI and NIH/NHLBI R01 HL076486-01 to 03, Wilson, PI) for the development and study of a coil-tipped magnetic catheter system, as described above.

## Authors’ contributions

LM and SH drafted the initial manuscript and figures. MS and MW critically edited and expanded upon the initial manuscript. All authors read and approved the final manuscript.
